# Neuroplasticity and immune system are related to altered grey matter networks: a cohort study in sporadic Alzheimer’s disease

**DOI:** 10.1093/braincomms/fcag257

**Published:** 2026-07-08

**Authors:** Diederick M de Leeuw, Flora H Duits, Ellen Dicks, Eleonora M Vromen, Charlotte E Teunissen, Frederik Barkhof, Wiesje M van der Flier, Pieter Jelle Visser, Betty M Tijms

**Affiliations:** Alzheimer Center Amsterdam, Neurology, Vrije Universiteit Amsterdam, Amsterdam UMC Location VUmc, 1081HV Amsterdam, The Netherlands; Amsterdam Neuroscience, Neurodegeneration, 1081HV Amsterdam, The Netherlands; Alzheimer Center Amsterdam, Neurology, Vrije Universiteit Amsterdam, Amsterdam UMC Location VUmc, 1081HV Amsterdam, The Netherlands; Amsterdam Neuroscience, Neurodegeneration, 1081HV Amsterdam, The Netherlands; Alzheimer Center Amsterdam, Neurology, Vrije Universiteit Amsterdam, Amsterdam UMC Location VUmc, 1081HV Amsterdam, The Netherlands; Department of Neurology, Mayo Clinic, Rochester, MN 55905USA; Alzheimer Center Amsterdam, Neurology, Vrije Universiteit Amsterdam, Amsterdam UMC Location VUmc, 1081HV Amsterdam, The Netherlands; Amsterdam Neuroscience, Neurodegeneration, 1081HV Amsterdam, The Netherlands; Amsterdam Neuroscience, Neurodegeneration, 1081HV Amsterdam, The Netherlands; Department of Laboratory Medicine, Neurochemistry Lab, Amsterdam UMC, Vrije Universiteit Amsterdam, 1081HV Amsterdam, The Netherlands; Department of Radiology and Nuclear Medicine, Amsterdam UMC, Vrije Universiteit, 1081HV Amsterdam, The Netherlands; Queen Square Institute of Neurology and Centre for Medical Image Computing, University College London, WC1N 3BG London, UK; Alzheimer Center Amsterdam, Neurology, Vrije Universiteit Amsterdam, Amsterdam UMC Location VUmc, 1081HV Amsterdam, The Netherlands; Amsterdam Neuroscience, Neurodegeneration, 1081HV Amsterdam, The Netherlands; Alzheimer Center Amsterdam, Neurology, Vrije Universiteit Amsterdam, Amsterdam UMC Location VUmc, 1081HV Amsterdam, The Netherlands; Amsterdam Neuroscience, Neurodegeneration, 1081HV Amsterdam, The Netherlands; Alzheimer Center Amsterdam, Neurology, Vrije Universiteit Amsterdam, Amsterdam UMC Location VUmc, 1081HV Amsterdam, The Netherlands; Amsterdam Neuroscience, Neurodegeneration, 1081HV Amsterdam, The Netherlands

**Keywords:** CSF proteomics, Alzheimer’s disease, grey matter networks

## Abstract

Grey matter network topology is altered in Alzheimer’s disease and these alterations are related to cognitive decline. Understanding the biological underpinnings of loss of brain connectivity may provide insights into mechanisms related to developing Alzheimer’s dementia (i.e. dementia A+). We investigated which biological processes as measured in CSF proteomics were associated with loss of brain connections across the Alzheimer’s disease continuum. We included 347 individuals with abnormal CSF amyloid [mean age ± standard deviation (SD) 66 ± 8; 98 cognitively unimpaired—A+, 88 mild cognitive impairment—A+, 161 dementia A+] and 146 cognitively unimpaired individuals with normal CSF amyloid (mean age ± SD 62 ± 8) and available T1w MRI-scans and CSF proteomic data (3097 proteins using tandem mass tag spectrometry) from the Amsterdam Dementia Cohort. We used an automated pipeline to construct grey matter networks from 3D-T1 sequences and for each network, calculated the small-worldness coefficient, which we previously found to be robustly related to cognitive decline. Linear models were applied to test associations between CSF protein levels and connectivity measures using an interaction term for clinical stage while controlling for connectivity density, age and sex. We validated our results in data from the Alzheimer’s disease Neuroimaging Initiative (ADNI). Pathway enrichment analysis was performed for proteins associated with loss of brain connectivity (*P* < 0.05) using the Gene Ontology database. Individuals across the Alzheimer’s disease continuum had lower small-worldness coefficients compared with controls (ANOVA *P* < 0.001). In amyloid positive individuals, higher levels of 222 proteins and lower levels of 482 proteins were associated with lower small-worldness coefficients and were enriched for innate immune system and neuroplasticity pathways, respectively. Stratified for disease stage, most protein associations with lower small-worldness coefficients were found in mild cognitive impairment A+ (*n* = 527 proteins) and dementia A+ (*n* = 799 proteins) with considerable overlap (*n* = 239 proteins). Proteins in these stages were enriched for complement activation and synaptic integrity. In cognitive unimpairment A+, we found proteins enriched for processes involved in apoptosis. We did not find any enriched biological processes in controls. Repeating analyses in ADNI indicated that similar biological processes were associated with altered grey matter network connectivity. Higher CSF levels of proteins involved in immune responses and lower levels of proteins related to neuroplasticity were associated with lower small-worldness coefficients across the Alzheimer’s disease continuum. This suggests that preserving cognitive function in the presence of amyloid and prevention of dementia A+ may require therapies that strengthen synapses and targets the innate immune system in addition to amyloid and tau.

## Teaser

De Leeuw *et al*. report that small-worldness values of grey matter networks decrease across the Alzheimer's continuum, which was associated with low CSF levels of proteins related to neuroplasticity, and high levels of proteins related to immune response.

## Graphical Abstract

**Graphical Abstract:**
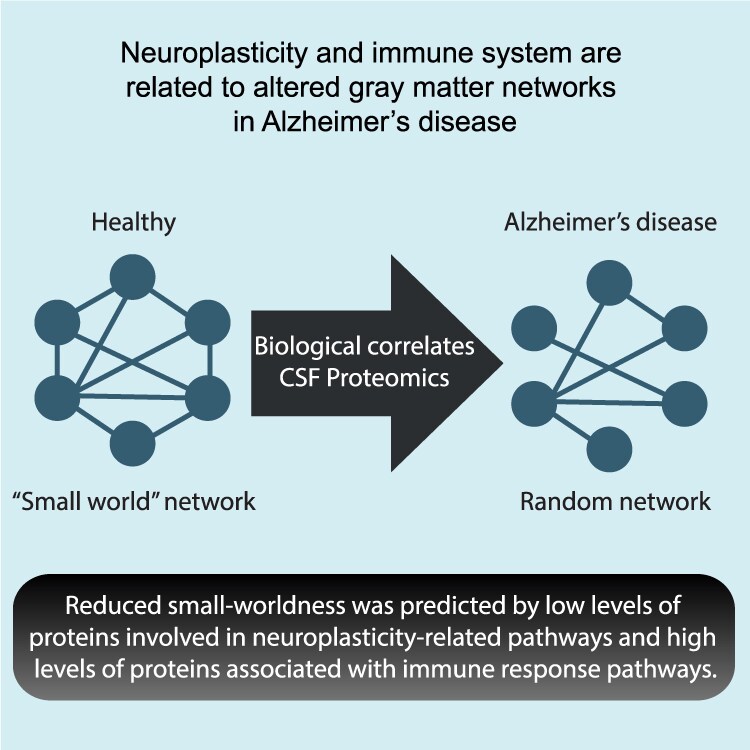

## Introduction

Alzheimer disease (AD) is the most common cause of dementia. The aggregation of amyloid into plaques is one of the first pathophysiological changes in the brain, which starts when cognition is still intact, about 15 years before dementia onset.^1^ Still, rates of decline in individuals with abnormal amyloid (A+) from intact cognition to mild cognitive impairment (MCI A+) and eventually to dementia and further on in the disease vary greatly between individuals, and the factors influence decline remain largely unclear.^2–10^ One factor related to rate of decline is the loss of grey matter network organization.^11–14^ Abnormal grey matter network (GMN) measures can already be observed in the earliest stages of the disease (i.e. when amyloid aggregation has already started but cognition is still intact, CU A+).^14–16^ CU A+ individuals who have abnormal GMN measures are at increased risk of clinical progression to MCI.^14^ Also, individuals with MCI A+ and abnormal GMN measures are nearly twice as likely to progress to dementia within 2 years as compared to individuals with normal GMN measures.^11,14,16,17^ Furthermore, these GMN measures explain variability in rates of decline in addition to markers related to tau tangles [i.e. phosphorylated tau (pTau) and total tau (tTau)].^12,13,17,18^ Possibly, treatments to improve GMN integrity might slow disease progression, and for this it is important to improve understanding of the biological mechanisms related to GMN integrity.

One approach to study biological mechanisms in patients is through analyses of protein levels in the cerebrospinal fluid (CSF). Taking a targeted approach, we recently observed in autosomal dominant AD that loss of GMN integrity as reflected by decline of small-worldness coefficient was related to higher CSF levels of proteins reflecting loss of axonal integrity and synaptic integrity (NfL, SNAP25, Neurogranin) as well as with YKL-40, a protein that might be involved in astrocytic reactivity and extracellular tissue remodelling (YKL-40).^19^ It remains unclear, however, to what such processes may also play a role in loss of GMN integrity as occurring in sporadic AD. Furthermore, it is now possible to measure many more proteins through untargeted mass spectrometry approaches. Using this approach, we and other previously found that distinct biological processes are involved in AD,^20–22^ including processes related to axonal integrity and inflammation, as well as other aspects of synaptic connectivity and more detailed information on the innate immune system such as the complement pathway. These processes are known to influence neuronal connectivity, and so alterations in those processes could be hypothesized to influence loss of GMN integrity in AD.^23–27^

In the current study we examined this hypothesis by testing which CSF protein levels, measured with Tandem Mass Tag (TMT) mass spectrometry, were related to lower GMN small-worldness coefficients in 347 individuals across the AD spectrum and 146 controls. We repeated our analyses in the ADNI cohort that had CSF proteomic measures available from platforms utilizing Multiple Reaction Monitoring (MRM) mass spectrometry and Rules-Based Medicine (RBM) multiplex immunoassays.

## Methods

### Consent statement

All participants or surrogates provided written informed consent.

### Participants

We selected individuals from the Amsterdam Dementia Cohort^28,29^ who were cognitively unimpaired, amyloid negative controls or amyloid positive individuals across the clinical spectrum (i.e. CU A+, MCI A+ and dementia A+) with available CSF proteomics and T1w MRI scans at baseline. All individuals visited our memory clinic at the Alzheimer centre, Amsterdam UMC, between November 2003–2019. They underwent extensive cognitive screening often including a neuropsychological test battery, lumbar puncture and MR imaging. Results are discussed in weekly multidisciplinary meetings in order to reach a consensus clinical diagnosis according to diagnostic and research guidelines of all major neurodegenerative diseases.^30–41^ The local institutional review board approved the procedures for the ADC and all patients provided written informed consent for use of their clinical data and biospecimens for research purposes.

### Replication cohort

We used the Alzheimer’s Disease Neuroimaging Initiative (ADNI) for replication analyses. ADNI is an ongoing longitudinal research cohort under the direction of Principal Investigator Dr. Michael W. Weiner. The main objective of ADNI has been to develop improved techniques that will lead to uniform acquisition of multisite and longitudinal MRI, PET as well as other biological and clinical data to track the development of MCI and early Alzheimer’s disease. More information about the ADNI is described in more detail at: http://adni.loni.usc.edu/. All ADNI participants provided written informed consent for study participation and data reuse. The regional ethics committees granted their approval in terms of ethics. Data for the current study were gathered between October 2005 and April 2013.

### MRI acquisition and preprocessing

T1-weighted scans were performed using previously published standardized protocols on 1, 1.5 or 3 T scanners in ADC and 1.5T or 3T scanners in ADNI.^28,42^ Statistical Parametric Mapping (SPM12, https://www.fil.ion.ucl.ac.uk/spm/software/spm12/) running in MATLAB (v2021a) was used to segment all scans into grey matter (GM), white matter, and CSF. To decrease the number of datapoints, the GM segmentations were resliced into 2 × 2 × 2 mm isotropic voxels. The sum of the GM, white matter, and CSF volumes was used to calculate the total intracranial volume (TIV). Hippocampal GM volume estimates were obtained using the automated anatomical labelling atlas. All GM segmentations were visually checked for quality.

### Single-subject GM networks

**Figure 1:**
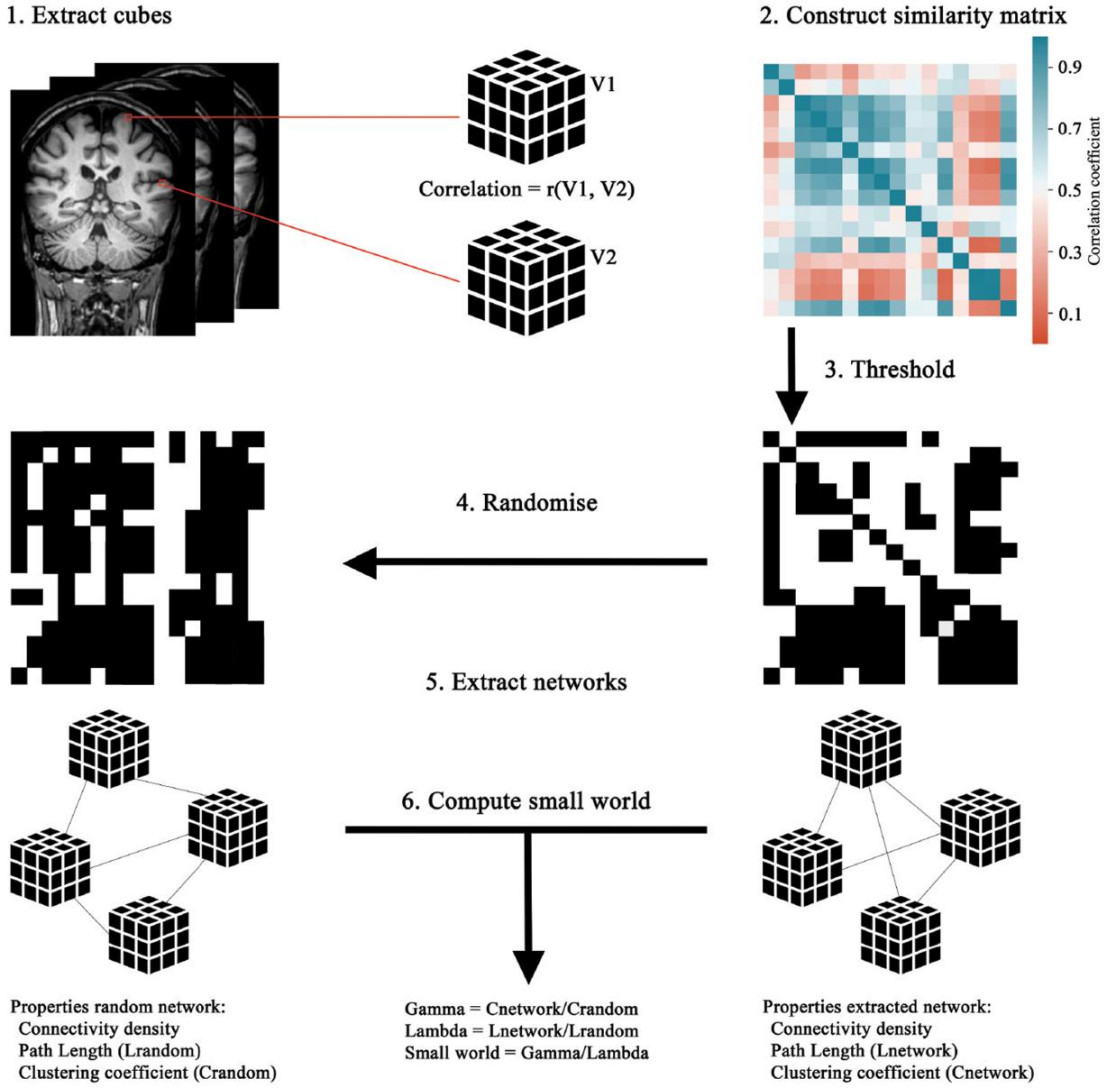
**Grey matter network processing pipeline.** Automated pipeline for computing single subject grey matter networks from T1-weighted MR images.

Single-subject GM networks were constructed from the GM segmentation maps using an automated pipeline (Fig. 1) as described in the publicly available MATLAB scripts: https://github.com/bettytijms/Single_Subject_Grey_Matter_Networks and in more detail in Tijms *et al*.^43^ For each individual’s scan, a network was constructed from native space GM segmentations. First, nodes were defined as cubes consisting of 3 × 3 × 3 voxels (6 mm × 6 mm × 6 mm) using an atlas-free approach. These nodes preserve the three-dimensional structure and so provide information on both GM intensity as well as the spatial relationships between voxels. Next, the structural similarity between each cube was computed with Pearson correlations across their corresponding voxels. Structurally similar regions in the cortex may not be rotationally aligned and so similarity values between cubes were also assessed by rotating one cube at angles with multiples of 45° over all axes in order to find the highest correlation value with a target cube throughout the curved cortex. Next, we determined for each network a threshold to only include connections with a *P* < 0.05 corrected for multiple comparisons using a permutation approach.^44^ Then, we calculated the small-worldness coefficient (σ) for each individual whole-brain GM network which reflects the optimal equilibrium between information segregation and integration of a network. The small-worldness coefficient is defined by the ratio between normalized clustering coefficient (γ) and normalized path length (λ). In short, γ measures how a network's clustering coefficient (the percentage of neighbours that are also neighbours of one another at a certain node) differs from a random network. Similarly, λ measures the deviation of the path length of a network (shortest path length between all pairs of nodes in the network) from a random network. In order to compute γ and λ, we divided the clustering coefficient and path length with the average of the corresponding measures obtained from five randomized reference networks that had the same size and degree distribution as the patient level network.^45^ All network measures were computed on whole brain networks with scripts from the Brain Connectivity Toolbox (https://sites.google.com/site/bctnet/),^46^ modified for large-scale networks. Small-worldness coefficient (SW) were used for further analyses as this measure has shown to be strongly associated with cognitive decline due to AD in previous work.^47^ Finally, we standardized SW values with the mean and standard deviation (SD) values of the control group in order to aid interpretation of effect sizes with protein levels (see next sections).

### CSF analysis

For ADC participants, CSF samples were obtained by lumbar puncture using a 25-gauge needle and syringe between the L3/L4, L4/L5, or L5/S1 intervertebral space, and collected in polypropylene tubes and processed as previously described.^28^ Amyloid β1–42 (Aβ42), phosphorylated tau_181_ (*P*-tau) and total tau (t-tau) concentrations were determined with enzyme-linked immunosorbent (ELISA) assays from Innotest (β-amyloid1-42, hTAU-Ag and PhosphoTAU-181p, Fujirebio, Ghent, Belgium). The previously published drift-corrected cut-offs Aβ42 < 813 pg/mL and t-tau >375 pg/mL were used to determine amyloid and tau abnormality. CSF proteomics was performed with liquid chromatography tandem mass spectrometry (LC-MS/MS) in an untargeted manner, based on tandem mass tag (TMT) labelling with 16-plexing, using a high pH reverse phase HPLC for peptide prefractionation, as described in detail previously.^48^ We used reference channels to normalize peptide relative abundances between TMT plex experiments, according to standard procedures.^49–51^ CSF was collected in polypropylene tubes after lumbar puncture with an atraumatic needle in ADNI. Aβ42, *P*-tau, and t-tau were measured using the INNO-BIA AlzBio3 kit on the Luminex xMAP platform.^52^ Cut-offs were <192 pg/mL for Aβ42 and >93 pg/mL for t-tau.^53^ T-tau was used over *P*-tau due to broader availability, with a strong correlation between both markers (ADNI: *r* = 0.65, *P* < 0.001; ADC: *r* = 0.95, *P* < 0.001). Proteomics analyses were performed with Multi Reaction Monitoring (MRM) targeted mass spectroscopy and Rules Based Medicine (RBM). Additional information on protein assessment and quality control is described at http://adni.loni.usc.edu/data-samples/biospecimen-data/. We used the finalized, quality-controlled ‘normalized intensity’ data from Spellman *et al*.^54^ (for a thorough explanation of the normalization process, see the ‘Data Primer’ page at adni.loni.ucla.edu for the ‘Biomarkers Consortium CSF Proteomics MRM dataset’). In total, 3863 proteins were detected in ADC and 449 proteins in ADNI. We included proteins in our analysis when detected in at least 50 of individuals, resulting in 3097 unique proteins in ADC and 198 unique proteins in ADNI. Prior to further analysis, all protein and protein fragment values were first normalized using the control group's mean and SD values.

### Statistical analysis

We compared clinical characteristics of the clinical groups (i.e. controls, CU A+, MCI A+ and dementia A+) with ANOVAs for continuous variables with a normal distribution and a Kruskal-test for variables with a skewed distribution. A Chi-square test was used to compare categorical variables and a Fisher’s exact-test for non-normal categorical variables. *Post hoc* analyses were performed where appropriate.

For each protein (predictor), we performed linear regression analysis to test if the CSF levels were related to small-worldness coefficients (outcome), while correcting for potential influence of age, sex, clinical stage and connectivity density. In addition, scanner model was added as a covariate (as multiple scanners were used) and we repeated analyses including APOE genotype as another covariate. We extracted main effects of protein levels from these models (model 1; Supplementary Table 1). We repeated this model with gamma and lambda as outcome measures in order to assess whether the main results are majorly driven by one or both of these properties on which the small-worldness coefficient is calculated (Supplementary Table 5). As a second linear regression analysis (model 2; Supplementary Table 1) we included an interaction term of protein level × diagnostic group, to study whether the associations were dependent on diagnostic group as well as to study the stage specific associations.

Next, for the total group as well as each diagnostic group separately, we performed biological pathway analyses for proteins that showed a significant association with the small world measure. We used a liberal threshold of *P* < 0.05 as we were interested in exploring which biological processes are related to grey matter networks. Pathway enrichment analyses were performed with GO for biological processes^55^ using the Panther tool, and *P* values were corrected for multiple testing with the false discovery rate procedure. All analyses were performed with R, version 4.3.3 (29 February 2024)—‘Angel Food Cake’.

## Results

### Participant demographics

**Figure 2:**
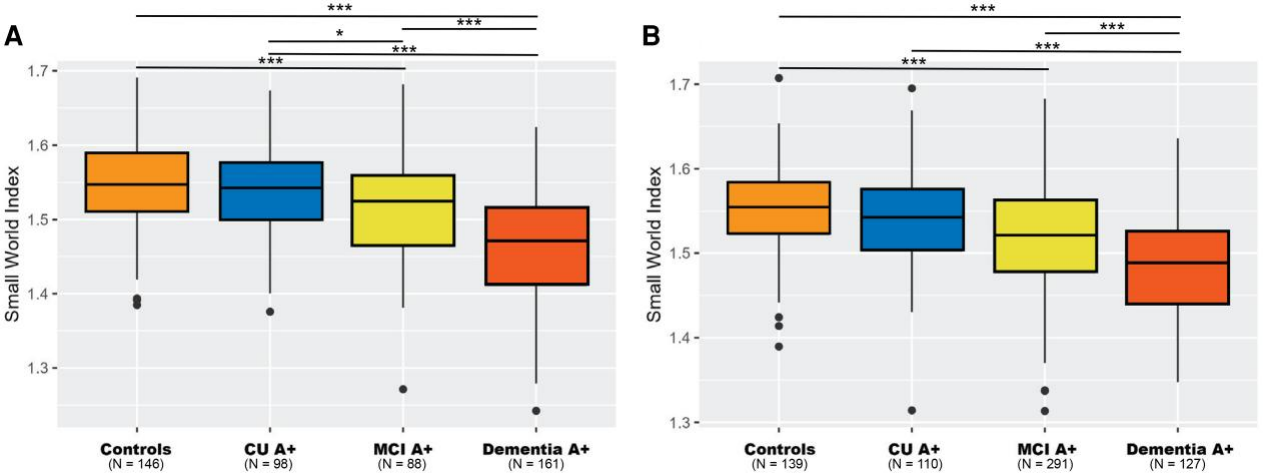
**Small-worldness across the AD clinical spectrum.** (**A**) Small-worldness coefficient across the AD clinical spectrum in ADC and (**B**) small-worldness coefficient across the AD clinical spectrum in ADNI. Differences were tested with ANOVA. Statistical significance is shown with an asterisk: *P* < 0.05*, *P* < 0.01**, *P* < 0.001***.

In total, 493 individuals were included from the ADC [mean ± SD age 64.7 ± 7.1 years, including 214 (43%) women, and 236 (48%) subjects that were A+T+, Table 1]. The ADNI validation cohort included 667 participants [mean ± SD age 73.5 ± 6.8 years, 314 (47%) female, 279 (42%) A+T+, Table 1]. In both cohorts, connectivity density was lower in MCI A+ and Dementia A+ compared with controls (ADC: *p*_controls-MCI A+_ = 0.01 and *p*_controls-Dementia A+_ <0.001; ADNI: *p*_controls-MCI A+_ <0.001 and *p*_controls-Dementia A+_ <0.001); and CU A+ in ADC (both *P* < 0.001). Similarly, small-worldness coefficients were lower with worse clinical stage (ADC: *p*_controls-MCI A+_<0.001, *p*_controls-Dementia A+_ <0.001, *p*_CU A+-MCI A+_ = 0.04, *p*_CU A+-Dementia A+_ < 0.001 and *p*_MCI A+-Dementia A+_ < 0.001; ADNI: *p*_controls-MCI A+_ < 0.001, *p*_controls-Dementia A+_ < 0.001, *p*_CU A+-Dementia A+_ < 0.001 and *p*_MCI A+-Dementia A+_ < 0.001; Fig. 2).

**Table 1:** Demographics of study participants in ADC and ADNI

	ADC						ADNI					
	Controls	CU A+	MCI A+	Dementia A+	Overall	*P* value	Controls	CU A+	MCI A+	Dementia A+	Overall	*P* value
*n*	146	98	88	161	493		139	110	291	127	667	
Age in years, mean ± SD	61.5 ± 8.4^a,b,c,d^	66.5 ± 8.0^a,b^	66.5 ± 7.8^a,c^	65.6 ± 7.1a,d	64.7 ± 7.1	<0.001	72.4 ± 5.6^a,b^	74.9 ± 6.1^a,b^	73.2 ± 7.2^a^	74.3 ± 7.4^a^	73.5 ± 6.8	0.02
Sex, female (%)	55 (38%)	53 (54%)	32 (36%)	74 (46%)	214 (43%)	0.07	67 (48%)	63 (57%)	122 (42%)	62 (49%)	314 (47%)	0.09
Connectivity density, mean ± SD	16.8 ± 1.1^a,c,d^	17.0 ± 1.2^a,e,f^	16.3 ± 1.1^a,c,e^	16.2 ± 1.2^a,d,f^	16.6 ± 1.2	<0.001	18.1 ± 1.1^a,c,d^	17.8 ± 1.1^a^	17.7 ± 1.1^a,c^	17.5 ± 1.1^a,d^	17.8 ± 1.1	<0.001
Gamma, mean ± SD	1.71 ± 0.09^c,d^	1.69 ± 0.08^e,f^	1.66 ± 0.10^c,e^	1.59 ± 0.09^a,d,f^	1.66 ± 0.10	<0.001	1.71 ± 0.07^c,d^	1.69 ± 0.08^e,f^	1.67 ± 0.08^c,e^	1.62 ± 0.08^a,d,f^	1.67 ± 0.08	<0.001
Lambda, mean ± SD	1.10 ± 0.02^c,d^	1.10 ± 0.01^e,f^	1.10 ± 0.01^c,e^	1.09 ± 0.01^d,f^	1.10 ± 0.02	<0.001	1.10 ± 0.01^c,d^	1.10 ± 0.01^e,f^	1.09 ± 0.01^c,e^	1.09 ± 0.01^d,f^	1.09 ± 0.01	<0.001
Small world index, mean ±SD	1.55 ± 0.06^c,d^	1.54 ± 0.06^e,f^	1.51 ± 0.07^c,e,g^	1.46 ± 0.07^d,f,g^	1.51 ± 0.07	<0.001	1.55 ± 0.05^c,d^	1.54 ± 0.06^f^	1.52 ± 0.06^c,g^	1.48 ± 0.06^d,g^	1.52 ± 0.06	<0.001
Biomarker profile (%)												
A−T−	146 (100%)	0 (0%)	0 (0%)	0 (0%)	146 (30%)		139 (100%)	0 (0%	0 (0%)	0 (0%)	139 (21%)	
A+T−	0 (0%)	45 (46%)	29 (33%)	37 (23%)	111 (23%)		0 (0%)	80 (73%)	137 (47%)	32 (25%)	249 (37%)	
A+T+	0 (0%)	53 (54%)	59 (67%)	124 (77)	236 (48%)		0 (0%)	30 (27%)	154 (53%)	95 (75%)	279 (42%)	

*P*-value < 0.05 for a: comparing same cognitive and amyloid group between ADC and ADNI, b: comparing controls and CU A+ in same dataset, c: comparing controls and MCI A+ in same dataset, d: comparing controls and dementia A+ in same dataset, e: comparing CU A+ and MCI A+ in same dataset, f: comparing CU A+ and dementia A+ in same dataset, g: comparing MCI A+ and dementia A+ in same dataset.

### CSF protein level associations with small-worldness coefficient values

**Figure 3:**
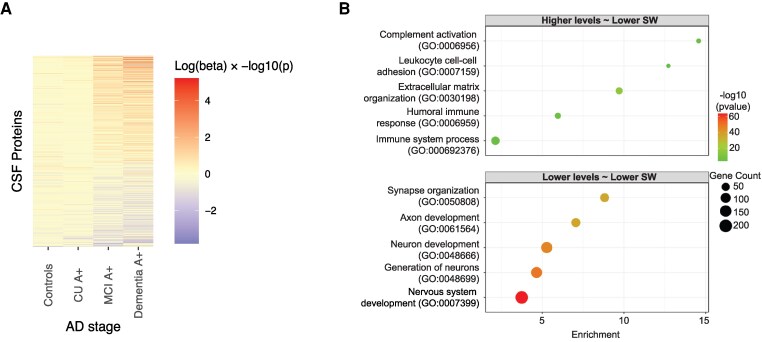
**CSF proteomic associations with small-worldness.** (**A**) Heatmap of CSF proteins levels (*n* = 3097) associated with SW values across the AD clinical spectrum in ADC as tested with linear regression. (**B**) Gene Ontology (GO) biological pathways associated with lower SW using PANTHER (see Supplementary Table 2 for all pathways).

**Figure 4:**
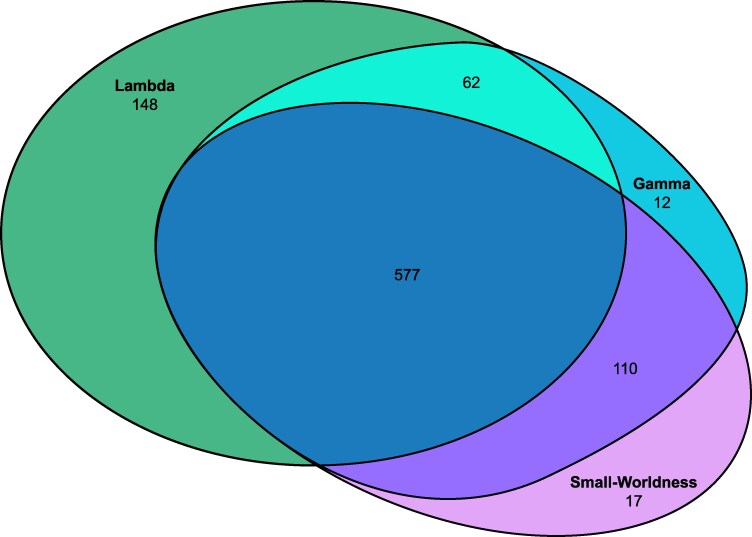
**Overlap of protein associations across network measures.** Overlap of CSF proteins associated with small-worldness coefficient, gamma and lambda. Each circle contains the total number of all proteins found to be significantly associated with small-worldness coefficient, gamma and lambda. Overlapping circles contain number indicating how many proteins were shared between network measures.

We tested cross-sectional associations of CSF protein levels and small-worldness coefficients. In ADC, we found a total of 704 proteins (23% of 3097 total proteins; Fig. 3A) that were associated with lower small-worldness coefficients (Supplementary Tables 1 and 4). Lower levels of 482 out of 704 proteins were associated with lower small-worldness coefficients and included NPTX2 (*β* = 0.013 ± 0.003; *P* < 0.001), VGF (*β* = 0.011 ± 0.003; *P* < 0.001) and APP (*β* = 0.01 ± 0.003; *P* < 0.001). These proteins were involved in various neuroplasticity mechanisms such as neuron development (GO:0048666; p_FDR_ = 1.45E−46) and synapse organization (GO:0050808; p_FDR_ = 1.48E−36; Fig. 3B). Still, the list of 482 proteins also contained some proteins implicated in inflammatory processes such as CX3CL1 (*β* = 0.008 ± 0.003; *P* < 0.01) and SIRPA (*β* = 0.005 ± 0.003; *P* = 0.04). Higher levels of 222 of the 704 proteins such as GPNMB (*β* = −0.009 ± 0.003; *P* < 0.01) and NEFM (*β* = −0.013 ± 0.003; *P* < 0.001) were associated with lower small world values and were mainly related to immune-related responses such as humoral immune response (GO:0006959; p_FDR_ = 1.45E−06) and complement activation (GO:0006956; p_FDR_ = 7.49E−06). In ADNI, a total of 37 proteins (19% of 198 total proteins) were found to be associated with lower small-worldness coefficient, of which 24 (65%) overlapped with the proteins found in ADC. Lower levels of 19 out of 37 proteins tested were related to lower small world index values, and these also included NPTX2, NPTXR, VGF and APP (Supplementary Table 1). These proteins were related to neuroplasticity processes (Supplementary Table 2). Higher levels of 18 out of 37 proteins such as NEFL (*β* = −0.008 ± 0.002; *P* < 0.001) and GFAP (*β* = −0.006 ± 0.003; *P* < 0.05) were associated with lower small world values. This group of proteins was involved in processes related to the immune system, like regulation of immune system process (GO:0002682; p_FDR_ = 7.19E−03). The majority of all 704 significantly associated proteins showed overlap with proteins associated with gamma and lambda (93% and 77% respectively, Fig. 4). Interestingly, we found 148 unique associations of CSF proteins with lambda. These proteins were enriched for axonal processes (e.g. axon development (GO:0061564), p_FDR_ = 4.65E−03 and axonogenesis (GO:0007409), p_FDR_ = 0.03). These axonal processes appeared unique to lambda, as enrichment was not found in the protein set overlapping with both gamma and small-worldness.

**Figure 5:**
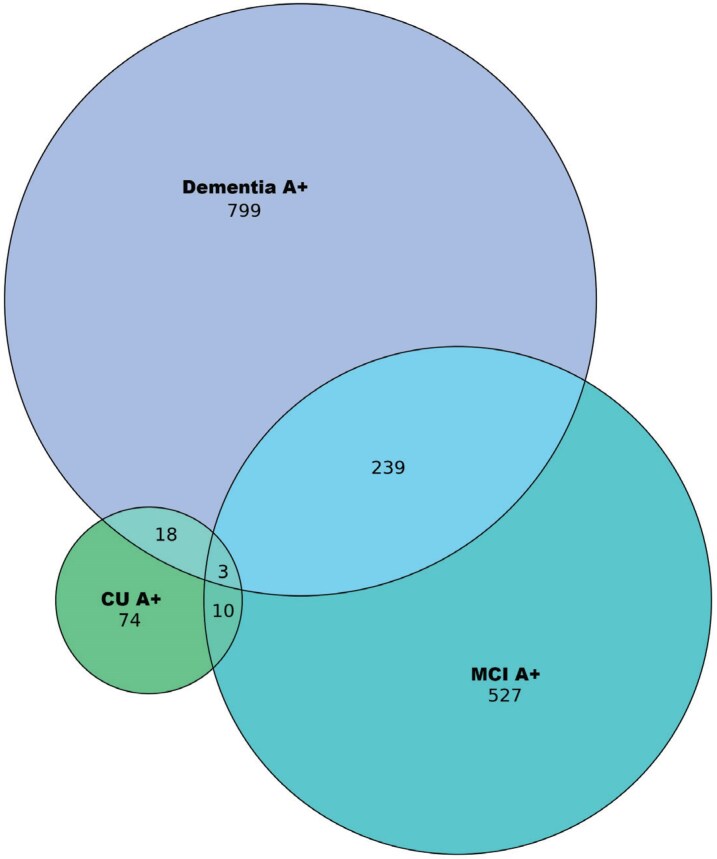
**Stage-specific overlap of protein–small-worldness associations.** Overlap of stage specific protein-small-worldness coefficient associations in all AD groups. Each circle contains the total number of all proteins found in that clinical stage. Overlapping circles contain number indicating how many proteins were shared between clinical stages. Note that controls are not included in this figure even though there was some overlap.

Next, we repeated analyses to study whether associations between CSF proteins and small-worldness coefficient were stage-specific. In the ADC, we observed that a total of 74 proteins were associated with lower small world values in CU A+ of which 48 proteins were specific to this preclinical stage (Fig. 5; Supplementary Table 1). Higher levels of 36 out 48 proteins were related lower small-worldness coefficient and were enriched for processes such as negative regulation of apoptotic process (GO:0043066; p_FDR_ = 4.51E−02). Lower levels of the remaining 12 out of 48 proteins were associated with lower small world, but these were not involved in any particular biological process according to go. In the prodromal stage, 527 proteins were associated with small world properties of which approximately half (*n* = 274, 52%) were specific to this stage (Supplementary Table 1). Out of these 274 proteins, higher levels of 129 proteins associated with lower small-worldness coefficient values. These 129 proteins were mainly related to immune responses such as complement activation (GO:0006956; p_FDR_ = 2.15E−05; Supplementary Table 3) and immunoglobulin mediated immune response (GO:0016064; p_FDR_ = 1.29E−04). Lower levels of the remaining 145 of 274 proteins associated with lower small-worldness coefficient and were enriched for processes like regulation of cell communication (GO:0010646; p_FDR_ = 2.18E−05) and regulation of trans-synaptic signaling (GO:0099177; p_FDR_ = 2.23E−04). In dementia, we observed a total of 799 proteins to be associated with small-worldness coefficient of which the majority (*n* = 530, 66%) were specific to the dementia stage and 239 (34%) proteins overlapped with the proteins found in the prodromal stage (Fig. 5). Higher levels of 203 out of the 530 proteins associated with lower small-worldness coefficients and were most strongly related to immune system processes such as adaptive immune response (GO:0002250; p_FDR_ = 1.30E−07) and B-cell-mediated immunity (GO:0019724; p_FDR_ = 9.82E−07). Lower levels of 327 proteins associated with lower small-worldness coefficients and included APP (*β* = 0.02 ± 0.005; *P* < 0.001). These 327 proteins were involved in neuroplasticity processes like neurogenesis (GO:0022008; p_FDR_ = 1.89E−26), synapse organization (GO:0050808, p_FDR_ = 1.41E−18) and synapse assembly (GO:0007416; p_FDR_ = 1.12E−15). All associated GO terms can be found in Supplementary Tables 2 and 3.

In addition to stage-specific effects, we found that 289 protein associations had a significant interaction term indicating that relationships were different across clinical stages. These stage-dependent effects were most pronounced in contrasts involving MCI A+ and dementia, where protein levels frequently displayed stronger relationships with small world properties compared with earlier stages (i.e. controls versus MCI A+, controls versus dementia, CU A+ versus MCI A+ and CU A+ versus dementia; Supplementary Table 1). Conversely, three proteins (NPTX2, SCN3B and DIPK1C) showed to be early and sustained markers of the disease as they were associated to small world properties in all stages.

Finally, we repeated analyses for the control group only, to study if results were specific to AD pathology. In controls, 110 proteins were associated with the small-worldness coefficient, of which higher levels of 55 proteins and lower levels of 55 proteins were associated with lower small world values. Some of these proteins overlapped with proteins found in other stages (3 proteins in CU A+, 11 proteins in MCI A+ and 19 proteins in dementia; Supplementary Table 1). Yet, this particular group of proteins found in controls were not enriched for any biological processes.

Repeating analyses in ADNI, we partly replicated the results found in ADC. In CU A+ we found higher levels of 14 proteins out of the total 37 to be associated with lower small-worldness coefficient, though none of which overlapped with the CU A+ specific proteins found in ADC. These proteins were enriched for processes such as complement activation (GO:0006956; p_FDR_ = 4.28E−04). In MCI A+, six proteins were associated with small world properties and were not enriched for any biological processes. In dementia, 37 proteins were related to small-worldness coefficient of which four proteins were also found in ADC. Out of these, lower levels of 29 proteins were associated with lower small world values. These proteins were mostly related to neuroplasticity processes which were also found in ADC, including regulation of cell communication (GO:0010646; p_FDR_ = 3.06E−04) and regulation of trans-synaptic signaling (GO:0099177; p_FDR_ = 7.74E−03).

## Discussion

The main finding of this study is that loss of small world properties of grey matter networks in AD is associated with lower levels of proteins related to synaptic integrity and axonal structure, and with higher levels of proteins related to immune related processes. Most of the relationships we observed were specific to a clinical stage with the majority of associations found in the prodromal and dementia stages, and only a few proteins were related to lower small-worldness coefficients in the preclinical stage. Furthermore, most processes underlying breakdown in grey matter networks seemed specific for AD, rather than a general aging effect as we observed only limited associations of protein levels with GMN integrity in the control group. Together, these results indicate that loss of GMN organization is related to lower levels of proteins associated with synaptic and axonal structures, and higher levels of proteins related to immune activation.

In line with previous studies, we observed lower small-worldness coefficients in AD compared with controls.^11,12^ In addition, we show that CSF levels of established markers for AD such as tau and neurogranin are associated with lower small-worldness coefficient values. This aligns with the findings of a previous study by Vermunt and colleagues in ADAD and is consistent with other literature that links altered synaptic plasticity to AD and cognitive decline.^21,48,56^ We extend on this knowledge by studying the whole CSF proteome in sporadic AD in which we observe that lower levels of proteins involved in neural and synaptic plasticity are associated with lower small-worldness coefficient. For instance, the synaptic protein NPTX2, which has been consistently linked to AD and cognitive performance in previous studies,^57–62^ was found to be a sustained marker throughout the AD continuum in this study. It is noteworthy that we observed that synaptic and neural plasticity was equally captured by small-worldness coefficient as well as gamma, but lambda showed many unique associations which were enriched for axonal processes. Although lambda is a graph-theoretical measure derived from grey matter networks, it reflects the efficiency of communication across nodes via connections. In addition, it has been suggested that intracortical similarities might arise as a result of axonal connectivity that can influence morphological measurements of the cortex.^63–65^ The enrichment for axonal processes among proteins uniquely associated with lambda therefore provides a biologically intuitive link: proteins involved in axon development and axonogenesis may influence—or serve as markers of—disrupted long-range connectivity. This suggests that lambda may capture biological pathways related to axonal structure and function, complementing its role as a topological measure of network integration. Altogether, this supports the idea that alterations in GMN may reflect loss of synaptic connectivity. Still, longitudinal studies are required to further study this hypothesis with serial CSF proteomic measurements and MR images. Moreover, more mechanistic studies are necessary to further understand what the morphological similarity measurements of brain structural organization reflect. Possibly such studies may be complemented with post-mortem investigations into the questions whether brain areas that are anatomically connected or share similar cortical gene expression.

Next, we observed that higher levels of proteins related to the innate immune system, such as complement activation, were associated with lower small world values. Complement activation has previously been demonstrated to play a role in synaptic pruning^66–68^ and occurs during normal neuronal development when microglia prune immature synapses.^69,70^ In AD, it has been suggested that this pathway may be re-activated specifically in microglia near diffuse and neuritic plaques could lead to excessive synaptic pruning.^24,25,71^ In the present study, we found several proteins supporting this hypothesis such as GPNMB and CX3CL1 which are implicated in activated microglia and microglia-neuron communication.^72–74^ This is further supported by the observed association of lower small world values and lower levels of SIRPA which is considered to be involved in an anti-phagocytic signal for microglial pruning of synapses.^75,76^ Excessive pruning of synapses by microglia can lead to exacerbated synapse loss seen in neurodegeneration which has been shown in mouse models,^67,75,77,78^ possibly as a precursor of brain atrophy. A previous study shows supporting evidence for this in humans.^48^

Conversely, a study by Pereira *et al.*^79^ reported that microglial activation may have a protective role against tau accumulation in individuals without dementia and with evidence of AD pathology. Similarly, animal studies have shown that microglia could limit amyloid plaque formation during early stages of AD, but not in late disease stages.^80^ In line with these studies, we observed these processes in the prodromal and dementia stages of AD, but did not find any microglia-related enriched pathways in CU A+. This supports the hypothesis that microglia function as reflected in CSF protein levels might depend on clinical stage. Some studies have suggested that immune activation is different in men and women at risk for AD.^81–83^ Although, we have corrected our analyses for sex, this would be an important topic to study further in future research.

Some potential limitations to our study have to be noted. Although proteins and biological processes replicated in ADNI across the whole group, we could not replicate our stage specific findings. Proteomics measured in ADNI were based on a pre-selected panel of proteins, which have been previously implicated in AD, resulting in a lower number of proteins in the dataset. Moreover, many proteins in ADNI did not meet our inclusion criteria for analysis (i.e. measured in at least 50 individuals) which may have led to loss of power. Furthermore, in this study, we were limited to investigating cross-sectional associations. This leaves the biological processes which drive longitudinal GMN decline out of scope. Future studies should study the biological mechanisms driving grey matter connectivity loss in AD over time, which is data we are now prospectively collecting.

In summary, we found that across the clinical spectrum of AD, lower levels of CSF proteins associated with neuroplasticity and higher levels of proteins associated with immune responses are associated with connectivity loss, suggesting that these processes play an important role in AD specific deterioration of GMN organization. These disease stage-specific findings provide further evidence for the multifaceted pathologic processes that underlie AD, which will probably require tailored treatments or combination therapy.

## Supplementary Material

fcag257_Supplementary_Data

## Data Availability

The data from the ADC cohort that support the findings of this study are available from the corresponding author, upon reasonable request. The data from the ADNI cohort can be requested online at https://adni.loni.usc.edu/. All analyses were performed in R using packages *lme4*, *lmerTest* and *emmeans*.
