# Supplementary material for: Neuroplasticity and immune system are related to altered grey matter networks: a cohort study in sporadic Alzheimer’s disease
Source: Brain Commun. 2026 Jul 8;8(4):fcag257. doi: 10.1093/braincomms/fcag257 (PMC13373788; doi:10.1093/braincomms/fcag257)
Supplement: fcag257_Supplementary_Data [file fcag257_supplementary_data.zip › Supplementary_table_headings.docx]

**Supplementary table headings**

**Supplementary Table 1:** Associations of CSF protein levels and small world coefficient in ADC and ADNI. Model 1 showcases main protein effects regardless of clinical stage. Model 2 shows stage specific effects and contrast between clinical stages.

**Supplementary Table 2:** GO pathway enrichment analysis in ADC and ADNI.

**Supplementary Table 3**: GO pathway enrichment analysis for stage specific effects in ADC and ADNI.

**Supplementary Table 4:** Associations of CSF protein levels and small world coefficient in ADC. Model 1 showcases main protein effects regardless of clinical stage with and without APOE carriage as a covariate.

**Supplementary Table 5:** Associations of CSF protein levels and small world coefficient, gamma and lambda in ADC respectively. Model 1 showcases main protein effects regardless of clinical stage.
